# A Correction

**Published:** 1860-02

**Authors:** 


### Editorial.

### A CORRECTION.

There are some intimations and expressions in an article on "dental advertising," by Dr. C. C. Dills, in the last number of the Register, which require a little correction. In that article the statement is made that the movement which was suggested in the November number of the Register, on page 243, is simply an embodiment of refined dental advertising, and that it is to be carried out by "urging these productions upon the country press for gratuitous publication."

Everybody knows what is the aim and intention of the ordinary, *egotistic* and *bombastic* style of advertising. Egotism characterizes them all. "Dr. B. can do any thing that anybody else can, and does it in a superior style, he also does some things that nobody else can, *and he insures all his work*, and besides he defies all competition, and has availed himself of every possible opportunity and facility for obtaining a knowledge of his profession."

Now here is the marrow and essence of quack advertising. Now the most superficial observer will see at a glance the nature, aim and intentions of such assertions. The sole object is to induce the people to patronize the *advertiser*.

Now, a statement (and it is not material how full) of what the *science* and *art* of a profession enable its members to do for suffering and marred humanity, is altogether a different thing, and entirely admissible when a want of intelligence upon the part of the people renders it necessary. Now in regard to the popular essays referred to, it is the intention to teach the real importance and value of the teeth, the means to be employed for retaining them in a healthy condition, and to direct attention to the practices and agencies by which they are injured and destroyed, and last of all, to direct attention to the proper means for restoration to health when they have been diseased. Preservation first, and after this restoration.

If such articles savor of quack advertisements, then we are utterly at fault in perception ; and the attempt to classify them as such is only excusable on the ground of inability to perceive the difference.

Modes of practice should scarcely, if at all, be touched upon in popular essays, yet it is occasionally admissible. The assertion that the publication of such essays, would be *urged* upon the country press, and sponged from them, is simply ridiculous in the extreme. Nobody will ever be *urged* to print them. They are prepared primarily and chiefly for the good of the people ; and all such articles, upon any subject, all editors who are true men, will be anxious to publish without *urging*. T.

